# TRIM16 Inhibits Inflammation by Interacting With and Ubiquitinating TRAF2 in a Colitis Model

**DOI:** 10.1111/jcmm.70917

**Published:** 2025-10-24

**Authors:** Dong‐Liang Li, Li Zhou, Bo Zhang, Shuai Wang, Qing‐Yu Liang, Qiu‐Hua Liu, Chen‐Jiang Qu, Liang Ji

**Affiliations:** ^1^ Department of General Surgery The First People's Hospital of Zhangjiagang City Suzhou China; ^2^ Center for Translational Medicine The First People's Hospital of Zhangjiagang City Suzhou China

**Keywords:** IBD, NF‐ĸB, TRAF2, TRIM16, ubiquitination

## Abstract

Tripartite motif 16 (TRIM16), an E3 ubiquitin ligase, plays crucial roles in regulating cell proliferation, differentiation, autophagy and immunity. Several studies have suggested that TRIM16 may function as an anti‐inflammatory factor in various diseases. However, the functional significance and regulatory mechanisms of TRIM16 in inflammatory bowel disease (IBD) have yet to be fully explored. This investigation examined the expression and underlying mechanisms of TRIM16 in both in vivo and in vitro models of inflammation. The expression of TRIM16 was significantly decreased in a dextran sulfate sodium (DSS)‐induced mouse colitis model and in lipopolysaccharide (LPS)‐stimulated RAW264.7 macrophages. Additionally, TRIM16 knockdown in LPS‐treated RAW264.7 cells resulted in increased mRNA levels of *iNOS*, *TNF‐α* and *IL‐6*, as well as in increased activation of the NF‐ĸB signalling pathway. Mechanistically, TRIM16 interacted with tumour necrosis factor receptor‐associated factor 2 (TRAF2), facilitating its ubiquitination, which in turn impeded NF‐ĸB signalling and reduced the expression of inflammatory mediators such as IL‐6. Our in vitro and in vivo findings highlight the critical role of TRIM16 in modulating the TRAF2/NF‐ĸB signalling pathway. Furthermore, the observed downregulation of TRIM16 was correlated with the onset of colitis, suggesting that TRIM16 may serve as a promising therapeutic target for both the prevention and treatment of this condition.

**Abbreviations**
DAI: disease activity index
DSS: dextran sulfate sodium
IBD: inflammatory bowel disease
IL‐6: interleukin‐6
iNOS: inducible nitric oxide synthase
LPS: lipopolysaccharide
TNF‐α: tumour necrosis factor‐α
TRAF2: tumour necrosis factor receptor associated factor 2
TRIM16: tripartite motif 16

## 1 Introduction

IBD is a chronic inflammatory disorder that affects the gastrointestinal tract and is characterised by its persistent, recurrent nature and considerable challenges to cure, thus markedly impacting the quality of life of afflicted individuals. The principal manifestations of IBD include Crohn's disease and ulcerative colitis [1], each exhibiting distinctions in the disease course, affected regions and underlying pathophysiology. Over the past few years, there has been a consistent increase in the global incidence of IBD, a trend that is also observed in East Asian countries, including China. This trend has posed considerable challenges to and pressures on health care systems, prompting intensified research efforts and the exploration of novel therapeutic approaches for this debilitating condition [2, 3]. Despite certain advancements in understanding IBD, its exact pathogenesis remains elusive. Current research indicates that a complex interplay of genetic predispositions, environmental factors, immune dysregulation, dysbiosis of the gut microbiota, and a compromised intestinal barrier collectively contributes to the onset and progression of IBD [4, 5]. Genetic factors may render certain individuals more susceptible to IBD, with environmental triggers potentially precipitating this disease in genetically susceptible individuals. Therapeutic strategies for IBD vary and include anti‐inflammatory drugs, immunosuppressive agents, biologics, and traditional Chinese medicine, among others [6]. However, approximately 40% of patients find these interventions suboptimal, occasionally requiring surgical interventions. The existence of refractory cases underscores the formidable challenge of effectively managing IBD. To improve the quality of life of IBD patients, there is an urgent need to further investigate disease pathogenesis and identify novel therapeutic targets. The comprehensive exploration of genetic factors, immune responses, the gut microbiota and intestinal barrier function holds promise for discovering more precise and personalised treatment modalities. Concurrently, the intensified development of innovative drugs and treatment approaches aims to increase the effectiveness of interventions, particularly for refractory cases. The integration of the strengths of modern and traditional medicine is anticipated to revolutionise the landscape of IBD treatment and offer patients better tools for managing and controlling this chronic condition.

Tripartite motif (TRIM) proteins constitute a class of proteins with E3 ubiquitin ligase activity, and this family comprises over 80 identified members. Owing to the shared molecular structure, the members of this protein family are also referred to as RBCC proteins. Typically, the molecular structure of these proteins includes one RING finger domain, 1 to 2 B‐box zinc‐binding domains, and one coiled‐coil domain [7]. Esposito et al. revealed that owing to the presence of the RING finger domain, the majority of TRIM family proteins actively participate in the ubiquitination process, thereby regulating biological processes, including cell proliferation, differentiation, autophagy and immune responses [8]. However, not all family members possess a RING finger domain; for example, the E3 ubiquitin ligase activity of the TRIM16 protein likely originates primarily from its B‐box domain [9]. As a tumour suppressor, TRIM16 plays a pivotal role in various cancers. Research suggests that TRIM16 effectively inhibits the invasion and metastasis of colorectal cancer by facilitating the degradation of Snail [10]. Additionally, research conducted by Wang et al. suggested that TRIM16 could competitively bind to p‐TAK1, promoting its ubiquitination and degradation and thereby alleviating lipid accumulation and inflammation in nonalcoholic fatty liver disease [11]. TRIM16 also interacts with NLRP3, promoting its ubiquitination and subsequent degradation and thereby attenuating inflammatory responses and apoptosis after myocardial ischaemia–reperfusion and decreasing the occurrence of myocardial infarction [12]. Recent studies have revealed that TRIM16 inhibits the phosphorylation of Prdx1, consequently mitigating pathological cardiac hypertrophy and heart failure [13]. Despite extensive research demonstrating the anti‐inflammatory properties of TRIM16 in various diseases, no study has explicitly elucidated its role in IBD. In‐depth exploration of the functional mechanism of TRIM16 in this context has the potential to provide novel perspectives and therapeutic strategies for the treatment of inflammatory bowel disease.

## 2 Materials and Methods

### 2.1 Antibodies and Reagents

Anti‐TRIM16 (cat. no. sc‐398,851), anti‐NF‐ĸB (cat. no. sc‐8008), anti‐p‐NF‐ĸB (cat. no. sc‐136,548), anti‐p‐IĸBα (cat. no. sc‐8404), anti‐IĸBα (cat. no. sc‐1643) and anti‐TRAF2 (cat. no. sc‐136,999) were purchased from Santa Cruz Biotechnology (Santa Cruz, CA, USA). Anti‐β‐actin (cat. no. 66009‐1‐Ig), anti‐TRAF2 (cat. no. 26846‐1‐AP), anti‐MYC (cat. no. 60003‐2‐Ig and cat. no. 16286‐1‐AP) and anti‐DYKDDDDK (cat. no. 20543–1‐AP and cat. no. 66008–4‐Ig) were purchased from Proteintech (Proteintech Group Inc., Wuhan, China). Anti‐TRIM16 (cat. no. ab72129) was purchased from Abcam (Cambridge, MA, USA). LPS (cat. no. L4391) was purchased from Sigma–Aldrich (St. Louis, MO, USA). DSS (cat. no. 216,011,080) was purchased from MP Biomedicals (USA).

### 2.2 Mice

Male C57BL/6 mice aged 6 weeks and weighing between 18 and 20 g were obtained from Airuide (Suzhou, China). The animals were kept under controlled aseptic conditions with a light/dark cycle of 12 h and had unrestricted access to food and water. All animal experiments were approved by the Animal Care and Use Committee at the Zhangjiagang First People's Hospital.

### 2.3 DSS‐Induced Colitis Model

The mice were administered 2.5% DSS in drinking water for 7 days. During this period, the mice were weighed every day, diarrhoea and blood in the stool were observed, and the disease activity index (DAI) score was determined (Table 1). All the mice were euthanized on Day 8, and colon tissues were collected.

**TABLE 1:** DAI score.

Score	Weight loss (%)	Diarrhoea	Blood in stool
0	0	Normal	Normal
1	1–5		
2	5–10	Loose stools	Slight bleeding
3	10–20		
4	> 20	Watery diarrhoea	Gross bleeding

### 2.4 Histological Staining

Fresh mouse colon tissue was fixed in 10% formalin, embedded in paraffin wax, and sectioned into 5‐mm slices. The sections were mounted on slides, dewaxed, stained with haematoxylin and eosin (H&E), and examined under a microscope by a professional pathologist.

### 2.5 Immunohistochemistry

The procedures for tissue fixation, paraffin embedding, and sectioning followed the aforementioned protocol. Following dewaxing, the sections were subjected to antigen retrieval by using the improved citrate antigen retrieval solution (P0083; Beyotime, Shanghai, China) at 100°C, followed by treatment with a 3% hydrogen peroxide solution to inactivate endogenous catalase. After being blocked with goat serum at room temperature for 1 h, the tissue sections were incubated overnight with a primary antibody at 4°C. Subsequently, the samples were incubated at room temperature for 30 min, after which a secondary antibody was applied. The DAB reagent (GK600710; GeneTech, Shanghai, China) was added to visualise the staining, and haematoxylin was used for counterstaining. Finally, a professional pathologist examined and evaluated the sections under a microscope.

### 2.6 Cell Lines and Cell Culture

RAW264.7 and HEK293T cells were procured from the Cell Bank of the Type Culture Collection of the Chinese Academy of Sciences, Shanghai, China. The cells were grown in DMEM (11,995,065; Gibco, CA, USA) supplemented with 10% FBS (10,270,106; Gibco, CA, USA), after which they were maintained in a 37°C incubator with 5% CO_2_. For LPS stimulation, RAW264.7 cells were treated with 50 ng/mL LPS for the specified times.

### 2.7 Lentiviral Cell Transfection

Lentiviruses containing specific short hairpin RNA sequences targeting TRIM16 (sh‐TRIM16‐1, sh‐TRIM16‐2, or sh‐TRIM16‐3; GeneChem, Shanghai, China) were transfected into RAW264.7 cells. The culture medium was replaced after 12 h, the infection efficiency was assessed under a microscope after 48–72 h, and the transfected cells were selected for further study with 5 μg/mL puromycin. The sequences of the shRNAs used are detailed in Table 2.

**TABLE 2:** Mouse TRIM16 shRNA target sequences.

No.	Target sequence (5′–3′)
TRIM16 shRNA‐1	CGCAAGTATAGGACCTCGAAA
TRIM16 shRNA‐2	GCTCGGTATCTATGTAAACTT
TRIM16 shRNA‐3	CGGGATGAGTTTCTTCAATAT

### 2.8 Plasmid Construction and Transfection

TRIM16 and TRAF2 were amplified via PCR and cloned into the pFLAG‐CMV‐2 and pCMV‐Myc vectors, respectively. The plasmid and LipofectamineTM 2000 reagent (Invitrogen, MA, USA) were diluted in serum‐free medium and mixed together. The resulting mixture was incubated for 5 min at room temperature and then added to HEK293T cells with high viability and a minimal passage number. After 4 h of incubation, the medium was replaced, and the cells were incubated at 37°C for 48–72 h before analysis. The primer sequences for the gene‐overexpressing plasmids are detailed in Table 3.

**TABLE 3:** Primers for overexpressing plasmid construction.

Gene	Primer sequence (5′–3′)
TRIM16 (flag)	Forward: GGTGAACTACTGTGAGGAGCA
TRIM16 (flag)	Reverse: CAGAAGGAAACCAAAGGGCTG
TRAF2 (myc)	Forward: GTAACCCGTTGAACCCCATT
TRAF2 (myc)	Reverse: CCATCCAATCGGTAGTAGCG
Ub (HA)	Forward: CTCCCAACAGACCTGTCTATAC
Ub (HA)	Reverse: CCATTGCACAACTCTTTTCTCA
Ub/K48R (HA)	Forward: GATCTTTGCTGGCAGGCAGCTGGAAGATG
Ub/K48R (HA)	Reverse: CATCTTCCAGCTGCCTGCCAGCAAAGATC

### 2.9 Immunofluorescence Staining

Equal numbers of untransfected RAW264.7 cells and cells stably expressing the TRIM16‐specific shRNAs were seeded onto slides. After a 24‐h incubation period, the cells were treated with LPS (50 ng/mL) for 30 min, fixed with 4% paraformaldehyde for 15 min, and permeabilized with 0.5% Triton X‐100 (1139ML100; BioFroxx, Germany) for 20 min. After three washes with 1× PBS, the cells were blocked with normal goat serum for 30 min. The slides were subsequently incubated overnight at 4°C with a primary antibody, followed by incubation for 30 min in the dark with a secondary antibody. Finally, the slides were sealed and observed via confocal microscopy.

### 2.10 Western Blotting

An appropriate volume of RIPA buffer (P0013B; Beyotime, Shanghai, China) was added to ground colon tissue or collected cells, followed by the addition of a PMSF solution (ST507; Beyotime, Shanghai, China). The cells were lysed on ice for 30 min and then centrifuged at 12,000 × ***g*** at 4°C for 20 min. The resulting lysate was subjected to SDS–PAGE, and separated proteins were transferred onto an NC membrane. Subsequently, suitable antibodies were used for WB, and protein expression was detected via an ECL system (ChemiQ 4800 mini, Bioshine).

### 2.11 Real‐Time PCR

Total RNA was extracted from appropriate samples with TRIzol (15,596,026; Invitrogen, MA, USA), and first‐strand cDNA was synthesised via the RevertAid first‐strand cDNA synthesis kit (K1622; Thermo Scientific, USA). qPCR analysis was subsequently conducted by using the SYBR Green Power master mix (4,367,659; Applied Biosystems, USA). The primers used are listed in Table 4.

**TABLE 4:** Primers for qPCR.

Species	Gene	Sequence (5′–3′)
Mouse	TRIM16	Forward: GGTGAACTACTGTGAGGAGCA
Mouse	TRIM16	Reverse: CAGAAGGAAACCAAAGGGCTG
Mouse	18S	Forward: GTAACCCGTTGAACCCCATT
Mouse	18S	Reverse: CCATCCAATCGGTAGTAGCG
Mouse	IL‐6	Forward: CTCCCAACAGACCTGTCTATAC
Mouse	IL‐6	Reverse: CCATTGCACAACTCTTTTCTCA
Mouse	TNF‐α	Forward: ATGTCTCAGCCTCTTCTCATTC
Mouse	TNF‐α	Reverse: GCTTGTCACTCGAATTTTGAGA
Mouse	iNOS	Forward: ATCTTGGAGCGAGTTGTGGATTGTC
Mouse	iNOS	Reverse: TAGGTGAGGGCTTGGCTGAGTG

### 2.12 Immunopurification and Mass Spectrometry

HEK293T cells transfected with FLAG‐TRIM16 or the control empty vector were lysed in lysis buffer containing a PMSF solution (ST507; Beyotime, Shanghai, China) on ice for 30 min. The resulting lysate was centrifuged, and the supernatant was incubated for 2 h at 4°C with an anti‐FLAG M2 agarose gel. Following centrifugation, we washed the agarose gel three times with 1× wash buffer to remove any residual supernatant. The eluted protein complexes were then subjected to sequencing and analysis by mass spectrometry. The lysis buffer, anti‐FLAG M2 agarose gel, and wash buffer were components of the FLAG immunoprecipitation kit (0000162024; Sigma–Aldrich, USA).

### 2.13 FLAG Immunoprecipitation Method

The cell lysis process followed the same protocol as described above, with the addition of a PMSF solution (ST507; Beyotime, Shanghai, China) to the lysis buffer. An appropriate amount of a melted and mixed anti‐FLAG M2 agarose gel was taken and washed five times with 1× wash buffer. Following centrifugation to remove the supernatant, the cell lysate was added, and the mixture was incubated overnight at 4°C. Subsequently, we removed the supernatant by centrifugation of the mixture and washed it three times with 1× wash buffer. Elution buffer was then added, and elution was carried out for 5 min at room temperature. Following the addition of 10× wash buffer (10 μL) and 2× sample buffer (20 μL), the mixture was boiled in a metal bath for 3 min at 100°C. Finally, the supernatant was centrifuged for WB detection.

### 2.14 A/G Bead Method

In accordance with the sample size, appropriate protein A/G beads (B23202; Selleckchem, USA) were selected and rinsed three times with lysis buffer (87,787; Thermo Scientific, USA). The beads were subsequently incubated with a primary antibody at room temperature for 2 h. Magnetic separation was carried out with a magnetic rack, the supernatant was discarded, and the beads were washed three times with lysis buffer. Next, the cell mixture was added, and the mixture was incubated overnight at 4°C. After magnetic separation, the supernatant was removed, and the beads were washed three times with lysis buffer. Finally, 5× SDS–PAGE sample loading buffer (P0015L; Beyotime, Shanghai, China) was added to the beads, and the mixture was boiled in a 100°C metal bath for 10 min. After magnetic separation, the supernatant was collected for WB detection.

### 2.15 Statistical Analysis

Statistical analysis was conducted via the Prism 8.02 software. The data are expressed as the mean ± standard deviation (*X* ± SD). An unpaired Student's *t* test was used for comparisons between two independent samples, whereas one‐way or two‐way analysis of variance (ANOVA) was employed for comparisons involving three or more groups, followed by the Bonferroni post hoc correction. Each experiment was independently repeated at least three times, with statistical significance defined as *α* = 0.05. Significance levels are indicated as *p < 0.05, **p < 0.01 and ***p < 0.001.

## 3 Results

### 3.1 Construction of a Murine Model of DSS‐Induced Colitis

**FIGURE 1:**
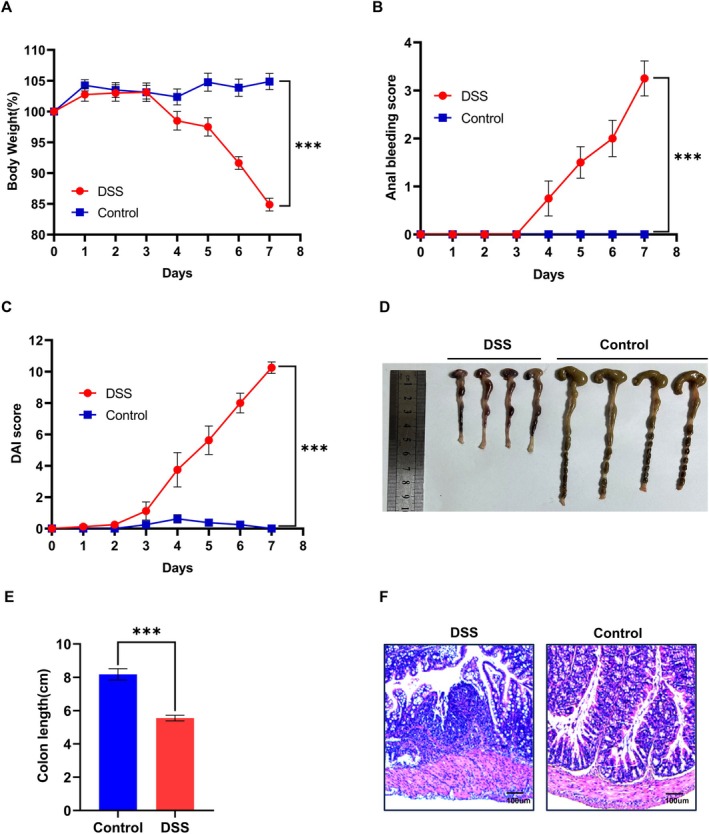
Successful construction of a mouse model of colitis. (A) Weight changes in the DSS‐induced and control groups (*n* = 8 per group). (B, C) Anal bleeding scores and DAI scores were examined from Days 0 to 7. (D, E) Macroscopic changes in the colons of mice that received 2.5% DSS treatment. The colon lengths were determined in both groups of mice. (F) H&E staining was performed on murine colon tissue sections from both groups on Day 8 after DSS treatment. The data are the means ± SDs. Statistical significance was determined via an unpaired Student's *t* test and two‐way ANOVA with the Bonferroni post hoc correction. ***p < 0.001.

Numerous studies have elucidated the pivotal role of TRIM16 as a regulator of inflammatory responses across a spectrum of diseases. Nonetheless, the anti‐inflammatory capacity of TRIM16 in IBD and its underlying mechanism remain unexplored. To gain a deeper insight into the biological function of TRIM16 in IBD, we employed a mouse model of DSS‐induced colitis. The results revealed that beginning on the fourth day, the body mass loss index (Figure 1A), anal bleeding score (Figure 1B), and disease activity index (DAI) score (Figure 1C) of the mice treated with DSS were significantly greater than those of the control group (mice that were dosed with distilled water alone). Compared with that in the control group, the colon length in the DSS‐treated group was significantly shorter (Figure 1D,E). In addition, through histological analysis, we detected significant inflammatory cell infiltration, destruction of the glandular structure, and damage to the mucosal layer, which are typical pathological features of colitis, in the colon tissue of the mice treated with DSS (Figure 1F). These data and the observed histological changes confirmed the successful establishment of a mouse model of DSS‐induced colitis and provided an important basis for the subsequent study of the role of TRIM16 in this model.

### 3.2 TRIM16 Expression Was Decreased in the Mouse Model of DSS‐Induced Colitis

**FIGURE 2:**
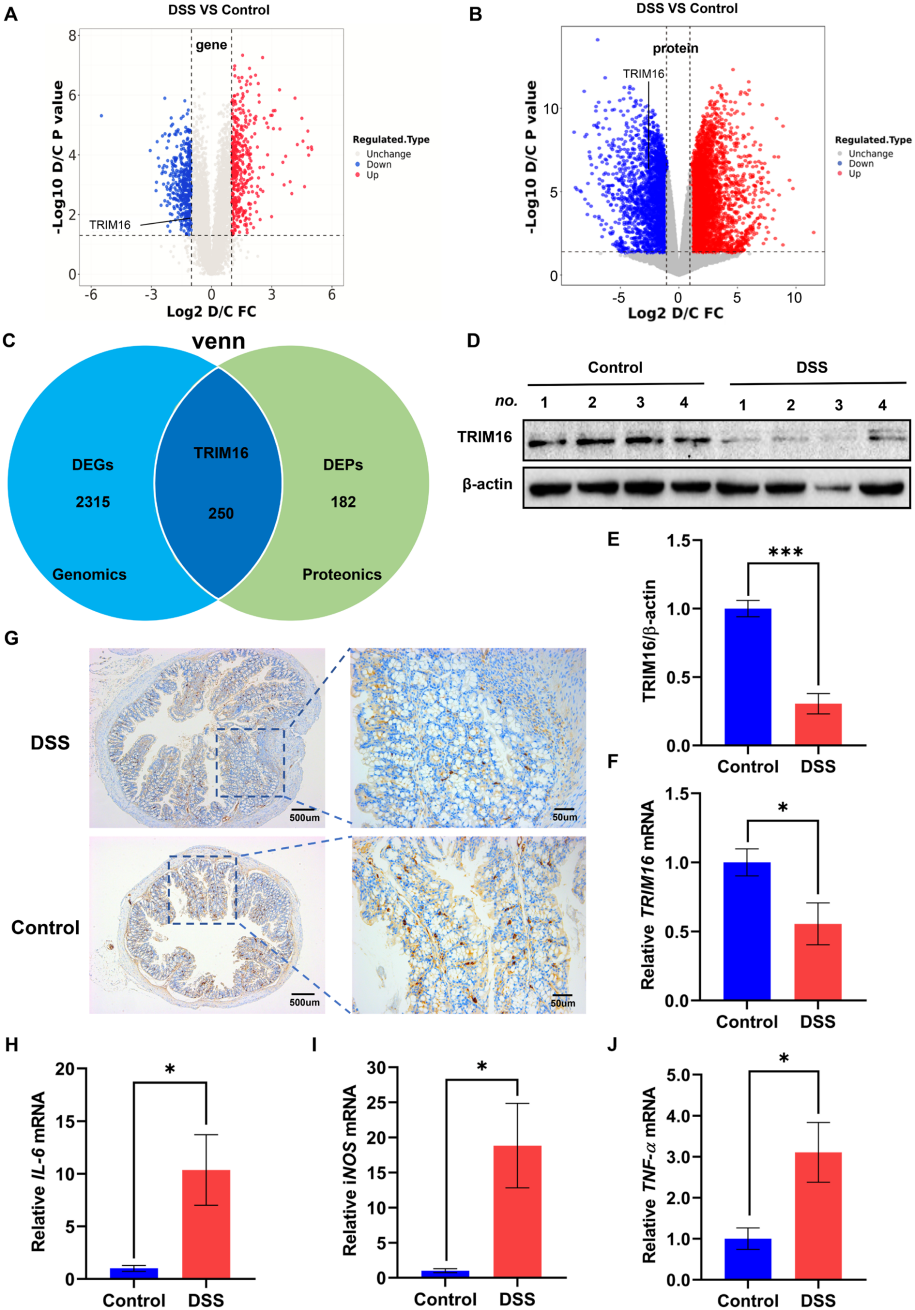
TRIM16 expression was downregulated in the colons of DSS‐treated mice. (A, B) Volcano plots of differentially expressed genes and proteins in colon tissue between DSS‐treated and control mice. (C) Venn diagrams of downregulated DEGs and DEPs between DSS‐treated and control mice. DEGs, differentially expressed genes. DEPs, differentially expressed proteins. (D, E) TRIM16 protein levels were determined by WB (*n* = 8). Quantification of the TRIM16 band intensity. (F) qPCR was used to evaluate *TRIM16* mRNA levels in colon tissues (*n* = 8). (G) Representative immunohistochemical staining results for TRIM16 in the colon tissue of mice (*n* = 8). (H–J) qPCR was used to evaluate *IL‐6*, *TNF‐α* and *iNOS* mRNA levels in colon tissues. The data are the means ± SDs. Statistical significance was determined via an unpaired Student's *t* test. *p < 0.05, ***p < 0.001.

To elucidate the key proteins involved in the inflammatory process of colitis, we employed proteomic and transcriptomic sequencing technologies to perform an extensive analysis of protein and mRNA expression profiles in colon tissue samples obtained from mice in both the DSS treatment and control groups. Compared with those in the control group, the proteomic data revealed a significant increase in the expression of 437 proteins and a decrease in the expression of 432 proteins in the DSS group. On the other hand, the transcriptomic results revealed 3794 genes whose expression was upregulated and 2565 genes whose expression was downregulated in the DSS group compared with the control group. On the basis of the results of many previous studies, we selected TRIM16, which plays an anti‐inflammatory role in a variety of diseases, from 250 downregulated genes for further study (Figure 2A–C). To verify this downregulation, we employed WB and qPCR. The expression of TRIM16 in colon tissues was compared between the two groups of mice. The results revealed a notable reduction in both the mRNA and protein levels of TRIM16 in the colon tissue of the DSS‐treated group compared with those in the control group (Figure 2D–F). Moreover, immunohistochemical staining analysis demonstrated a significant decrease in TRIM16 expression within colonic mucosal tissues of the mice treated with DSS compared with that in the control group (Figure 2G).

The development of colon inflammation is closely associated with the expression of intracellular inflammatory factors [14]. In our study, we utilised qPCR to examine the levels of inflammatory factors in the mouse model of acute DSS‐induced colitis. Our findings revealed a decrease in *TRIM16* mRNA expression in the DSS treatment group, while the mRNA expression levels of *IL‐6*, *TNF‐α*, *iNOS*, and other crucial inflammatory factors were significantly elevated compared with those in the control group (Figure 2H–J).

### 3.3 TRIM16 Expression Was Decreased in the LPS‐Stimulated RAW264.7 Cell Inflammation Model

**FIGURE 3:**
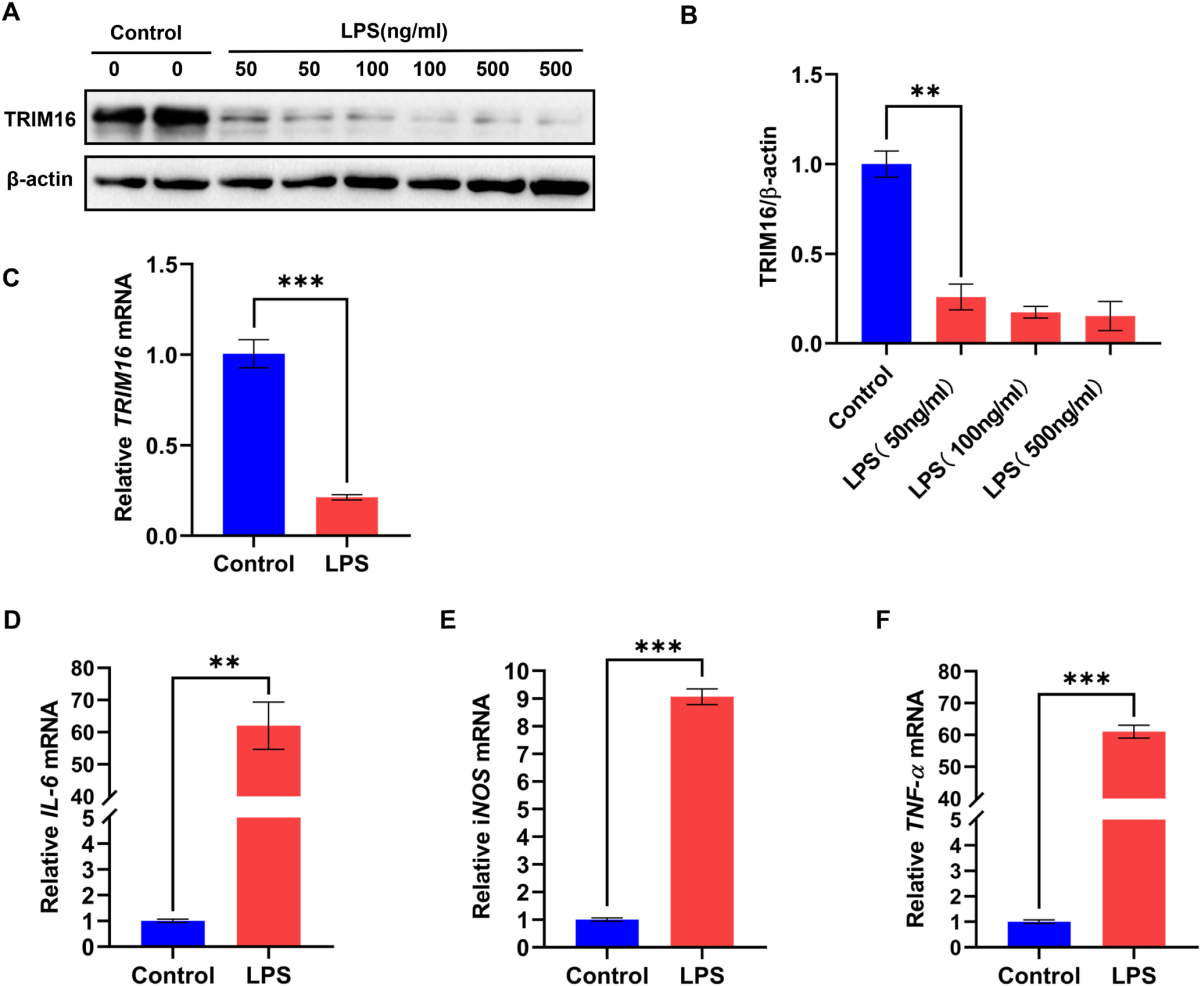
TRIM16 expression was downregulated in the LPS‐stimulated RAW264.7 cell inflammation model. (A, B) WB was used to assess the expression of TRIM16 in LPS‐stimulated RAW264.7 cells with different concentrations of LPS (50, 100 and 500 ng/mL). Quantification of the TRIM16 band intensity. (C) qPCR was used to evaluate *TRIM16* mRNA levels in LPS‐stimulated RAW264.7 cells (*n* = 3). (D–F) qPCR was used to evaluate the *IL‐6*, *TNF‐α* and *iNOS* mRNA levels in LPS‐stimulated RAW264.7 cells (*n* = 3). The data are the means ± SDs. Statistical significance was determined via an unpaired Student's *t* test; **p < 0.01, ***p < 0.001.

To delve deeper into the role of TRIM16 in inflammation, we reviewed the literature [14, 15] and investigated TRIM16 expression in an inflammatory environment in vitro using RAW264.7 cells as a model. To simulate inflammatory conditions, various concentrations (50, 100 and 500 ng/mL) of lipopolysaccharide (LPS) were used to stimulate RAW264.7 cells. After TRIM16 protein expression was analysed via WB, 50 ng/mL LPS was determined to be the optimal stimulation concentration (Figure 3A). The experimental data revealed that TRIM16 protein expression decreased significantly after treatment with 50 ng/mL LPS for 24 h compared with that in the control group without LPS treatment (Figure 3B). Similarly, through qPCR analysis, we confirmed that *TRIM16* mRNA expression in RAW264.7 cells significantly decreased following 12 h of stimulation with the same concentration of LPS (Figure 3C). Additionally, the levels of key inflammatory factors, such as *IL‐6*, *TNF‐α* and *iNOS*, were greater in the LPS‐stimulated group than in the control group according to the results of the qPCR analysis (Figure 3D–F). Based on these in vitro results and the aforementioned in vivo mouse model data, we tentatively concluded that downregulation of TRIM16 was closely associated with colitis pathogenesis.

### 3.4 Low Expression of TRIM16 Aggravated the Inflammatory Response in the LPS‐Stimulated RAW264.7 Cell Inflammation Model

**FIGURE 4:**
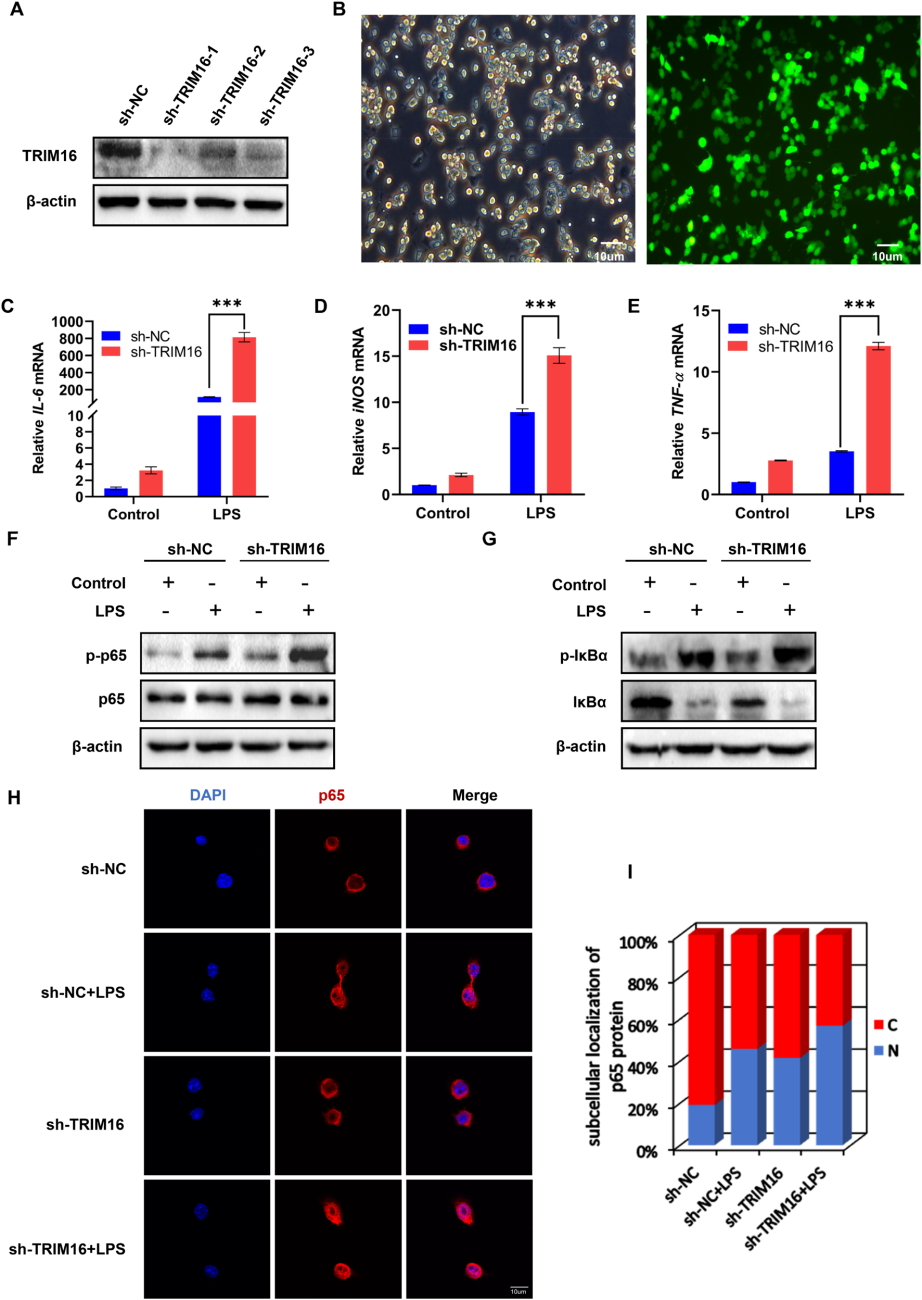
TRIM16 knockdown promotes inflammatory NF‐ĸB signalling activity in an LPS‐stimulated RAW264.7 cell inflammation model. (A, B) TRIM16 knockdown in the RAW264.7 cell line was achieved via the transfection with TRIM16‐specific shRNA1, shRNA2 or shRNA3 and confirmed via WB. (C–E) RAW264.7 cells transfected with sh‐TRIM16‐1 were stimulated with LPS, and the mRNA levels of *IL‐6*, *TNF‐α* and *iNOS* were analysed by qPCR (*n* = 3). (F, G) RAW264.7 cells were transfected with sh‐TRIM16‐1, then stimulated with LPS, and examined for the protein levels of p‐p65, p65, p‐IĸBα and IĸBα via WB. (H, I) RAW264.7 cells were transfected with sh‐TRIM16‐1, then stimulated with LPS, and immunostained with an anti‐p65 antibody. TRIM16 is labelled red, and the nuclei stained with DAPI are shown in blue. The percentages of cells with different subcellular localizations of the p65 protein were calculated. The data are the means ± SDs. Statistical significance was determined via one‐way or two‐way ANOVA with the Bonferroni post hoc correction. ***p < 0.001.

To better understand the regulatory role of TRIM16 in colitis, this study aimed to investigate the effect of reduced TRIM16 expression on inflammation via an in vitro inflammation model. In this study, the specific knockdown of TRIM16 in RAW264.7 cells was achieved by constructing lentiviral vectors containing three short hairpin RNA (shRNA) sequences targeting TRIM16 (labelled sh‐TRIM16‐1, sh‐TRIM16‐2 and sh‐TRIM16‐3). The transfection efficiency was confirmed via fluorescence microscopy, and the most effective knockdown sequence, sh‐TRIM16‐1, was selected via WB (Figure 4A,B). RAW264.7 cells transfected with sh‐TRIM16‐1 were stimulated with LPS, and the mRNA levels of *IL‐6*, *TNF‐α*, *iNOS*, and other major inflammatory factors were quantitatively analysed via qPCR. Statistical analysis revealed that the expression levels of these inflammatory markers were significantly greater in the TRIM16‐knockdown cell population than in the negative control group (Figure 4C–E).

The activation of the transcription factor nuclear factor‐kappa B (NF‐κB) pathway plays a pivotal role in the inflammatory response. To elucidate the influence of the TRIM16 protein on the NF‐κB signalling pathway, we initially evaluated the levels of p65 and its phosphorylated form p‐p65 in sh‐NC (control group) and sh‐TRIM16‐1 (experimental group) cells by WB. LPS stimulation led to increased p‐p65 protein levels in both groups, particularly in the sh‐TRIM16 group, suggesting that downregulation of TRIM16 might enhance the activation of the NF‐κB signalling pathway (Figure 4F). In addition, immunofluorescence staining was used to analyse the nucleolar accumulation of p65 following 30 min of LPS treatment in both groups of cells. The results indicated significant intracellular accumulation of p65 in both the sh‐NC and sh‐TRIM16‐1 groups following LPS induction. Notably, more pronounced nucleolar accumulation of p65 was observed in sh‐TRIM16‐1 cells than in control cells (Figure 4H,I). With respect to the regulatory effect of IκBα, the levels of both IκBα and its phosphorylated form, p‐IκBα, in sh‐NC and sh‐TRIM16‐1 cells treated with LPS were analysed by WB. The results demonstrated that LPS treatment resulted in a significant decrease in the level of IκBα protein and a relative increase in the level of p‐IκBα protein in sh‐TRIM16‐1 cells (Figure 4G). Taken together, these data support the hypothesis that TRIM16 serves as a potential regulator of the NF‐κB signalling pathway in inflammation models, suggesting its potential influence on the activation and function of NF‐κB through the modulation of p65 phosphorylation and IκBα expression.

### 3.5 TRIM16 Interacts With TRAF2

**FIGURE 5:**
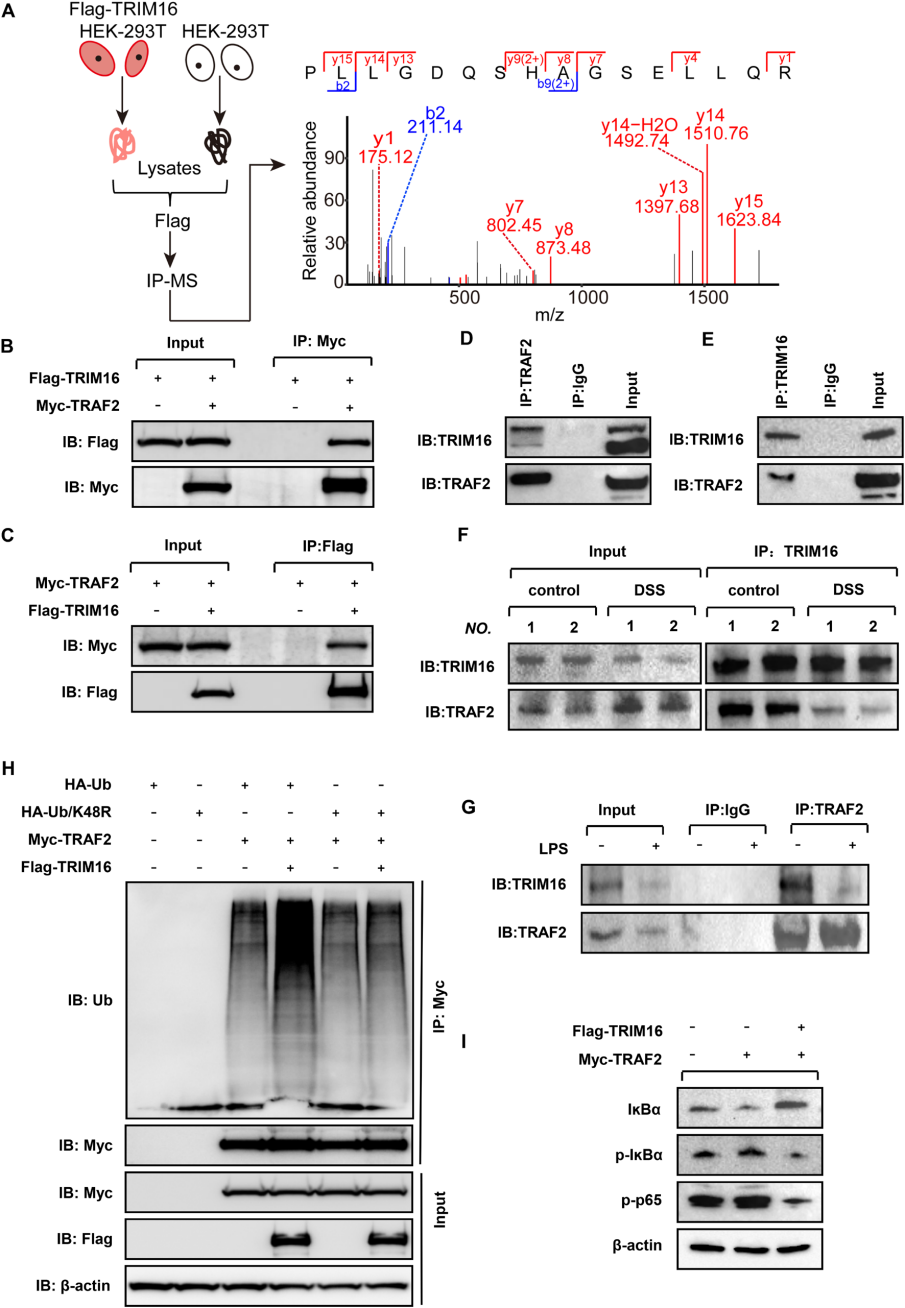
TRIM16 interacts with TRAF2 and regulates TRAF2 autoubiquitination. (A) Schematic representation of the screening for TRIM16‐binding proteins by IP‐MS. A secondary mass spectrum of peptides showing the identification of the TRAF2 protein. (B, C) Endogenous interaction of TRIM16 with TRAF2. Immunoprecipitation (IP) and immunoblot (IB) analyses of RAW264.7 cell lysates. (D, E) Exogenous interaction of TRIM16 with TRAF2. Immunoblot analysis of lysates after immunoprecipitation of HEK293T cells transfected with Flag‐TRIM16 and Myc‐TRAF2. (F) Interactions between TRIM16 and TRAF2 were analysed by IP in intestinal samples from mice treated with or without DSS. (G) LPS inhibits the interaction between TRIM16 and TRAF2. IP and IB analyses of lysates of RAW264.7 cells treated with LPS (50 ng/mL). (H) TRIM16 promotes the ubiquitination of TRAF2. HEK293T cells were transfected with the indicated plasmids for 50 h; the lysates were analysed via IP with an anti‐Myc antibody, and the ubiquitination of TRAF2 was examined via WB. (I) TRIM16 inhibits the activation of the NF‐ĸB signalling pathway by interacting with TRAF2. WB was used to assess the levels of IĸBα, p‐IĸBα and p‐p65 in HEK293T cells transfected with Myc‐TRAF2 and Flag‐TRIM16.

Considering the evident role of TRIM16 in regulating inflammation in vivo and in vitro, we next sought to investigate the mechanism by which TRIM16 inhibits inflammation in colitis. Immunoprecipitation coupled with mass spectrometry was employed to identify proteins that potentially interacted with TRIM16 in HEK293T cells transfected with the TRIM16‐expressing plasmid. The results revealed that TRIM16 could coprecipitate with many proteins, including tumour necrosis factor receptor associated factor 2 (TRAF2) (Figure 5A). TRAF2 is a key adaptor in the TNFR signalling complex that facilitates downstream signalling cascades, such as the activation of the NF‐κB signalling pathway [16]. Whether TRIM16 is involved in the activation of the NF‐κB pathway through its interaction with TRAF2 remains to be clarified. Therefore, we used plasmid transfection and immunoprecipitation to explore the interaction between TRIM16 and TRAF2 in exogenous and endogenous systems. First, the FLAG‐TRIM16 and MYC‐TRAF2 plasmids were cotransfected into HEK293T cells, followed by IP analysis of the lysates with an anti‐MYC or anti‐Flag antibody, and the protein expression of TRAF2 and TRIM16 was evaluated via western blotting. As shown in Figure 5B,C, many proteins were coimmunoprecipitated with TRAF2 or TRIM16. Similar results were observed in endogenous systems. Immunoprecipitation of TRAF2 in RAW264.7 cells with an anti‐TRAF2 antibody resulted in a significant amount of the coimmunoprecipitated TRIM16 protein. Similarly, immunoprecipitation of TRIM16 in RAW264.7 cells with an anti‐TRIM16 antibody resulted in a significant amount of the coimmunoprecipitated TRAF2 protein (Figure 5D,E). Collectively, these findings suggest that TRIM16 is capable of interacting with TRAF2. However, in the mouse model of DSS‐induced colitis, the interaction between TRIM16 and TRAF2 was weakened during DSS induction (Figure 5F). Consistently, we observed that LPS significantly diminished the endogenous interaction between TRIM16 and TRAF2 (Figure 5G). Emerging evidence suggests that TRIM proteins mediate K48‐ or K63‐ubiquitination in response to the activation of the NF‐κB signalling pathway. Therefore, we analysed the ubiquitination pattern of TRIM16, which is involved in the suppression of inflammation. The results showed that TRIM16 could increase TRAF2 ubiquitination through its interaction with TRAF2. Moreover, our study revealed that the ubiquitination involved K48‐linked ubiquitin, leading to the degradation of TRAF2 and inhibition of the occurrence and development of inflammation (Figure 5H). The FLAG‐TRIM16 and MYC‐TRAF2 plasmids were transfected into HEK293T cells separately or together, and the levels of p‐p65, p‐IκBα and IκBα were analysed to further determine whether TRIM16 regulates the NF‐κB signalling pathway through TRAF2. The results revealed that the protein levels of p‐p65 and p‐IκBα in HEK293T cells transfected with MYC‐TRAF2 were significantly greater than those in nontransfected HEK293T cells. However, the protein levels of p‐p65 and p‐IκBα in HEK293T cells transfected with FLAG‐TRIM16 and MYC‐TRAF2 were significantly lower than in those transfected with MYC‐TRAF2 only (Figure 5I). These results suggest that TRIM16 inhibits the activation of the NF‐κB signalling pathway through its interaction with TRAF2.

## 4 Discussion

In this study, we noted a significant reduction in the level of TRIM16 expression in the colon tissue of DSS‐treated mice compared with that in the control group. Similarly, the expression level of TRIM16 in LPS‐stimulated RAW264.7 cells was notably lower than that in the control group. Moreover, reduced TRIM16 expression markedly aggravated the LPS‐induced inflammatory response in RAW264.7 cells. We further demonstrated that TRIM16 was an inhibitor of the NF‐ĸB signalling pathway. Mechanistically, TRIM16, acting as a potential E3 ubiquitin ligase, interacted with TRAF2 to increase its ubiquitination, thereby inhibiting the NF‐ĸB signalling pathway. Our results clearly provide robust evidence for the role of TRIM16 as a protective agent in the pathogenesis of colitis.

Previous studies have shown that TRIM16 is associated with a variety of diseases, including cancer, such as neuroblastoma and melanoma [10, 17, 18, 19, 20]. As a cell‐protective factor, TRIM16 not only alleviated oxidative damage to human periodontal ligament stem cells by activating the PICOT/Akt/Nrf2 pathway [21] but also interacted with galectin‐3 to protect cells from lysosomal damage by activating selective autophagy [22]. In this study, transcriptomic and proteomic sequencing technologies revealed that the mRNA and protein expression levels of TRIM16 in the colon tissue of the mice in the DSS group were significantly lower than those in the control group. We subsequently confirmed these results via WB, PCR and immunohistochemistry. Additionally, the levels of IL‐6, TNF‐α, iNOS and other inflammatory factors were markedly elevated in the colon tissue of DSS‐treated mice compared with those in the control group. Therefore, we preliminarily suggest that the downregulation of TRIM16 is related to inflammation in DSS‐treated mice. Many TRIM proteins have been shown to be involved in inflammatory responses. In contrast to the proinflammatory effects of TRIM47 in vitro and in vivo [23], our findings revealed that the expression of TRIM16 was decreased in LPS‐stimulated RAW264.7 cells, whereas the mRNA levels of inflammatory factors significantly increased. Thus, we suggest that the downregulation of TRIM16 is associated with inflammation in LPS‐stimulated RAW264.7 cells. We subsequently observed that TRIM16 knockdown markedly intensified the inflammatory response in RAW264.7 cells. This outcome further validated the anti‐inflammatory effect of TRIM16.

The NF‐κB signalling pathway is an important intracellular signalling pathway that is involved in the regulation of biological processes such as inflammation, immune responses, cell proliferation and apoptosis. In a cell resting state, NF‐κB assumes an inactive status within the cytoplasm and binds to its suppressor IκB to form a stable p50‐p65‐IκB complex. The activation of this signalling pathway involves a complex mechanism, mainly through IκB kinase (IKK) complex‐mediated phosphorylation of the p65 subunit and IκBα, which promotes the nuclear translocation of the p65 subunit and leads to transcriptional regulation. Studies have shown that the inhibition of NF‐κB activation is an important therapeutic strategy for IBD [24]. In this study, we demonstrated that TRIM16 knockdown promoted the phosphorylation of p65 and IκB and enhanced the nuclear translocation of p65. These results imply that the downregulation of TRIM16 may activate NF‐κB signalling.

**FIGURE 6:**
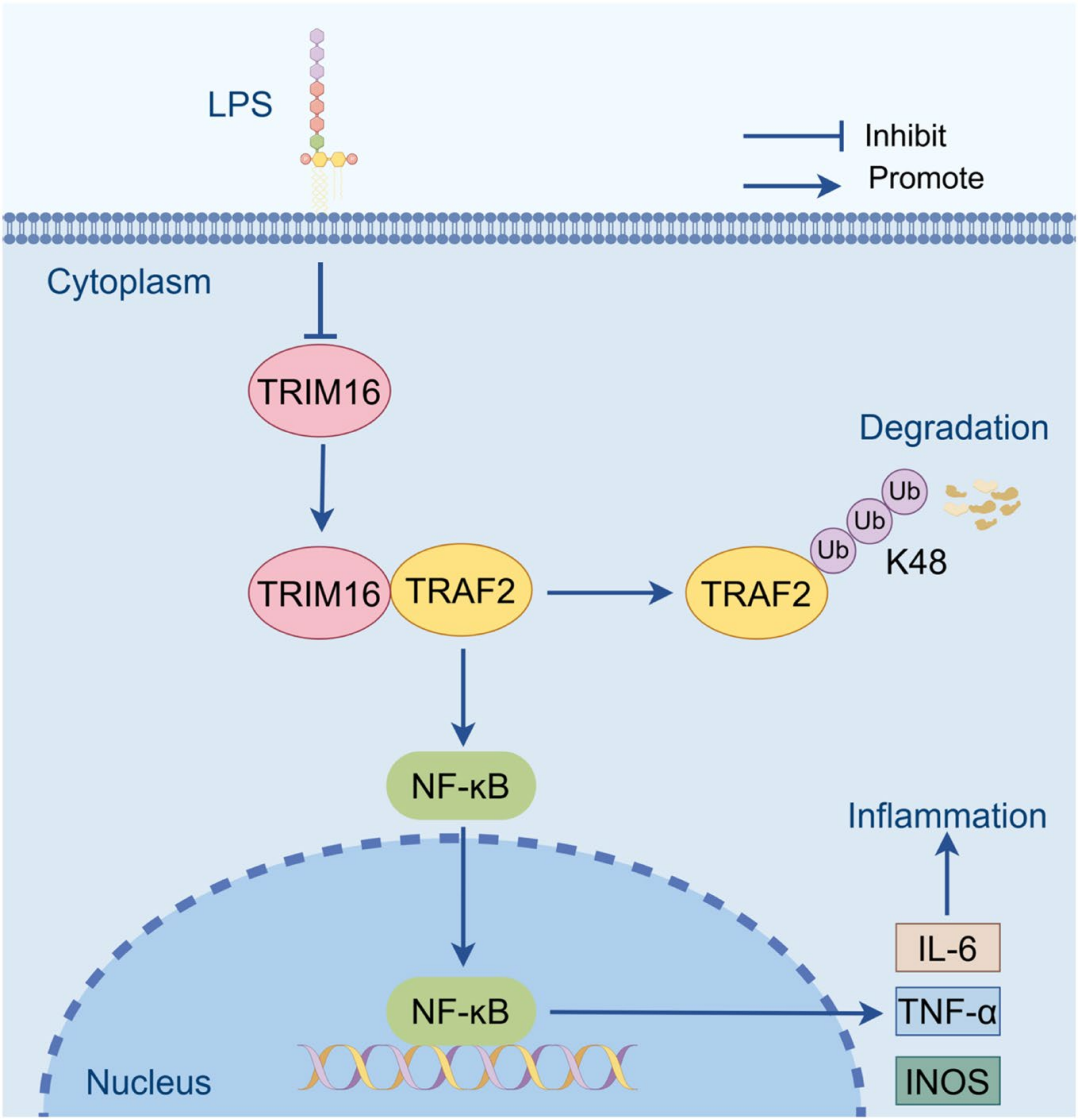
Schematic representation of the role of TRIM16 in response to LPS stimulation in macrophages. The figure was drawn by Figdraw.

TRAF2 represents a pivotal signal transduction protein within the TRAF family and exerts a significant influence on the classical NF‐κB pathway by governing TNF activation [25]. Upon TNF stimulation of TNFR1, TRAF2 is recruited to the appropriate receptor complex, where it mediates the activation of downstream signalling proteins, including NF‐κB and MAPKs [26, 27]. Previous studies have demonstrated a marked increase in the TRAF2 expression levels in colon tissues and serum samples obtained from individuals afflicted by IBD [28]. However, whether TRIM16 regulates the occurrence and development of colitis by interacting with TRAF2 and activating the NF‐κB signalling pathway remains to be clarified. Therefore, we used coimmunoprecipitation and mass spectrometry to verify this hypothesis. The results showed that TRIM16 and TRAF2 could interact in both endogenous and exogenous systems. As a protein with E3 ubiquitin ligase activity, TRAF2 can participate in the activation of the classical NF‐κB signalling pathway through K63‐linked ubiquitination and in the activation of the nonclassical NF‐κB signalling pathway through K48‐linked ubiquitination [25]. Studies have shown that TRIM family proteins can positively or negatively regulate NF‐κB signalling through ubiquitination. For example, TRIM25 interacts with TRAF2 and enhances the K63‐linked polyubiquitin chains attached to TRAF2 [29]. TRIM47 can activate the NF‐κB and MAPK signalling pathways by enhancing K63‐linked TRAF2 ubiquitination [7]. Moreover, it can form a complex with nuclear factor 90, thereby exerting antiviral innate immune responses via K48‐linked ubiquitination [30]. Recent studies have reported that TRIM16 mediates the ubiquitination of K48‐linked p‐TAK1, consequently impeding the activation of the JNK–p38 cascade, which is implicated in NASH progression [11]. Furthermore, we found that the TRIM16 interaction with TRAF2 increased the ubiquitination of TRAF2 and that the ubiquitination process was mediated by K48 linkages. The functional dichotomy between these regulatory mechanisms suggests an intricate balance in TRAF2 activity control, where different E3 ligases may fine‐tune inflammatory responses through distinct ubiquitination patterns. Furthermore, our findings complement recent work on OTUD7B, a deubiquitinase that stabilises TRAF2, revealing a yin‐yang relationship in TRAF2 homeostasis that may be crucial for maintaining intestinal immune balance [31]. In addition, we performed coimmunoprecipitation experiments both in vivo and in vitro in experimental colitis models. These experiments revealed that the downregulation of TRIM16 resulted in a reduced interaction with TRAF2. Finally, we found that the phosphorylation of IκB and p65 was increased in TRAF2‐transfected HEK293T cells. However, in HEK293T cells cotransfected with TRAF2 and TRIM16, the increases in the p‐IκB and p‐p65 levels were abolished. Together with our previous experimental findings, we propose that the downregulation of TRIM16 results in diminished TRAF2 ubiquitination at the K48 site, which consequently impedes TRAF2 degradation and augments the activation of the NF‐κB signalling pathway, ultimately contributing to the onset and progression of colitis (Figure 6).

Together, our results suggest that reduced expression of TRIM16 promotes inflammatory cell infiltration and activation of the TRAF2/NF‐ĸB inflammatory signalling pathway in colon tissue. Our study identified TRIM16 as a negative regulator of the pathogenesis of colitis, which promotes the ubiquitination of TRAF2 and can be used as a new target for the clinical prevention and treatment of colitis.

## Author Contributions

**Dong‐Liang Li:** conceptualization (lead), data curation (lead), formal analysis (lead), investigation (lead), methodology (lead), visualization (lead), writing – original draft (lead), writing – review and editing (lead). **Li Zhou:** conceptualization (lead), data curation (lead), formal analysis (lead), investigation (lead), methodology (lead), supervision (lead), validation (lead), writing – review and editing (lead). **Bo Zhang:** data curation (equal), investigation (equal), resources (equal), validation (equal). **Shuai Wang:** formal analysis (equal), investigation (equal), software (equal). **Qing‐Yu Liang:** data curation (equal), methodology (equal). **Qiu‐Hua Liu:** conceptualization (equal), resources (equal), supervision (equal). **Chen‐Jiang Qu:** conceptualization (equal), funding acquisition (equal), resources (equal), supervision (equal). **Liang Ji:** conceptualization (equal), funding acquisition (lead), project administration (lead), resources (lead), supervision (lead).

## Ethics Statement

The study was approved by the Animal Care and Use Committee at the Zhangjiagang First People's Hospital.

## Conflicts of Interest

The authors declare no conflicts of interest.

## Data Availability

The data that support the findings of this study are available from the corresponding author upon reasonable request.
